# Hepatic Arterial Vasodilation Is Independent of Portal Hypertension in Early Stages of Cirrhosis

**DOI:** 10.1371/journal.pone.0121229

**Published:** 2015-03-20

**Authors:** Miriam Moeller, Antje Thonig, Sabine Pohl, Cristina Ripoll, Alexander Zipprich

**Affiliations:** First Department of Internal Medicine, Martin Luther University Halle-Wittenberg, Halle (Saale), Germany; CIMA. University of Navarra, SPAIN

## Abstract

**Introduction:**

The compensatory increase in hepatic arterial flow with a decrease in portal venous flow is known as the hepatic arterial buffer response. In cirrhosis with elevated portal pressure, the vascular resistance of the hepatic artery is decreased. Whether this lower resistance of the hepatic artery is a consequence of portal hypertension or not remains unknown.

**Study Aim:**

The aim of the study was to investigate the hepatic arterial resistance and response to vasoconstriction in cirrhosis without portal hypertension (normal portal resistance).

**Methods:**

Cirrhosis was induced by CCl_4_-inhalation for 8 weeks (8W, normal portal resistance) and for 12–14 weeks (12W, elevated portal resistance). Bivascular liver perfusion was performed at 8W or 12W and dose response curves of methoxamine were obtained in the presence or absence of LNMMA (nitric oxide synthase blocker). Vascular resistances of the hepatic artery (HAR), portal vein (PVR) and sinusoids (SVR) were measured. Western Blot (WB) and Immunohistochemistry (IHC) were done to measure eNOS and HIF 1a expression.

**Results:**

HAR in both groups of cirrhotic animals (8W and 12W) were lower compared to controls. Dose response curves to methoxamine revealed lower HAR in both cirrhotic models (8W and 12W) regardless the magnitude of portal resistance. LNMMA corrected the dose response curves in cirrhosis (8W and 12W) to control. WB and IHC show increased protein expression of eNOS and HIF1a in 8W and 12W.

**Conclusion:**

Hepatic arterial resistance is decreased in cirrhosis independent of portal resistance. Vasodilation of the hepatic artery in cirrhosis seems to be influenced by hypoxia rather than increase in portal resistance. Nitric oxide is the main vasodilator.

## Introduction

The liver has a unique dual blood supply through the portal vein and the hepatic artery. The total blood supply to the liver is tightly regulated, so that changes in the portal venous blood flow are counteracted by opposite changes in the hepatic arterial flow. This phenomenon is known as the hepatic arterial buffer response [1]. For example, the postprandial splanchnic vasodilatation leads to an increase in portal venous blood flow and higher blood and oxygen supply through the portal vein. This is compensated immediately with a decrease in the hepatic arterial blood flow [2–5]. These abrupt changes are regulated locally by paracrine mediators with a short half life. Indeed, the vasodilator adenosine is produced locally in the normal hepatic circulation and has a central role in the regulation of the hepatic arterial buffer response [2,4], as shown by complete abolishment of the buffer response with adenosine blockade.

In cirrhosis, there is an increase in intrahepatic vascular resistance due to structural and dynamic changes [6–8]. This increase in the intrahepatic vascular resistance leads to an increase in portal pressure and a decrease in sinusoidal perfusion from the portal system. In rats with cirrhosis and ascites, the increase of the intrahepatic vascular resistance detected in the portal system is associated to a decrease in the hepatic arterial vascular resistance [9]. The mechanisms underlying this vasodilatation are associated to structural changes of the vessel wall itself (remodeling) and overexpression of two different vasodilators [10,11], namely nitric oxide and adenosine.

However, the regulation of the hepatic arterial flow in cirrhosis is not fully clarified. In the normal liver it is suggested that this buffer response allows maintenance of the total liver blood flow and a relatively stable liver oxygen supply [1,2]. In cirrhosis, the hepatic arterial vasodilatation that we had observed [10] could be explained according to two hypothesis. Firstly, the main drive could be the maintenance of the total hepatic blood flow to the liver. In this case the reduction in sinusoidal perfusion as a consequence of portal hypertension would be the stimulus to increase the arterial supply. This would be similar to what has been observed in the normal liver. In this case in cirrhosis without portal hypertension, no hepatic arterial vasodilatation should be detected. On the other hand, it could be that in cirrhosis the hepatic artery has an independent regulation, so that the vasodilation observed could be a consequence of other factors specific to the disease such as intrahepatic hypoxia, an early phenomenon in cirrhosis [12]. According to this latter hypothesis, hepatic arterial vasodilation should be detected independently of the presence of portal hypertension.

The first aim of the study was to investigate the hepatic arterial resistance in cirrhotic livers without portal hypertension and compare this to the known changes of the hepatic arterial resistance in cirrhotic livers with portal hypertension. The second aim of the study was to investigate possible mechanism that could be involved in changes of the hepatic artery in cirrhotic livers without portal hypertension including endothelial dysfunction, intrahepatic hypoxia and structural changes of the vessel wall.

## Methods

### Induction of CCl_4_-cirrhosis

Male Wistar rats underwent inhalation exposure of carbon tetrachloride (CCl_4_) three times a week. Phenobarbital (0.35 g/l) was added to the drinking water as described previously [9]. Treatment was given for 8 weeks (cirrhosis with normal portal resistance) and for 12 weeks (cirrhosis with elevated portal resistance). Hemodynamic perfusion experiment or tissue collection were performed 6 to 10 days after the last doses of CCl_4_ and phenobarbital.

### In situ rat liver perfusion

Rats were anesthetized with ketamine hydrochloride (Ketaset, Fort Dodge, USA; 100 mg/kg body wt) and xylazine (Rompum, Bayer, Germany; 40 mg/animal). A bivascular liver perfusion was performed as previously described [9,10]. Briefly, after the abdomen was opened loose ligatures were placed around the aorta cranial to the celiac artery, around the superior mesenteric artery immediately after branching from the aorta, and the aorta caudal to the mesenteric artery. Left gastric and splenic arteries were tied at their origin from the celiac artery and a loose ligature was placed around the esophagus. Left and right renal arteries as well as the gastroduodenal artery (branch of the common hepatic artery) were ligated. The bile duct was cannulated with a polyethylene tube (PE 10). The portal vein was then cannulated with a 14-gauge Teflon catheter and perfused with 30 ml/min of oxygenated (95% O_2_, 5% CO_2_) Krebs-Henseleit solution in a non-recirculating mode was started. The animal was sacrificed immediately by sectioning the caudal vena cava. Afterwards the aorta was cannulated with an 18-gauge Teflon catheter and the ligatures around the superior mesenteric artery and the esophagus were closed. The perfusion of the hepatic artery with 10 ml/min of oxygenated (95% O_2_, 5% CO_2_) Krebs-Henseleit solution in a non-recirculating mode was started. The tip of the catheter was placed close to the branch of the celiac artery and all ligatures around the aorta were closed. A 14-gauge catheter was introduced in the caudal vena cava and the thorax was opened.

In order to measure the sinusoidal pressure, a PE-50 catheter was guided from the right atrium, through the thoracic segment of the caudal vena cava into the left hepatic lobe and wedged in the hepatic vein. The ligature around the inferior vena cava was closed to secure the wedged catheter initiating the stabilization period.

During the stabilization and the experimental period the perfusion pressure of the portal vein and the hepatic artery were measured constantly using two independent physiological pressure transducers (MLT 844, AD Instruments, USA), respectively. The wedged pressure was measured during the experimental period using a third independent physiological pressure transducer (MLT 844, AD Instruments, USA). Before each experiment, all pressure measurement systems were calibrated with the zero point at the level of the hepatic hilum. Perfusion and sinusoidal pressure were continuously recorded using PowerLab 8/35 and Chart v5.5.4 program (AD instruments, USA). Throughout the whole perfusion period the perfusate was oxygenated using a silastic tubing lung interposed between the perfusate reservoir and the peristaltic pump and the perfusion flow was kept constant.

### Experimental design

The stabilization period was performed in a recirculating mode in absence or presence of the NO-production inhibitor L-NMMA (10^–4^ M; Sigma Chemicals Co., Germany). After the stabilization period the wedged catheter outflow was interrupted, allowing the measurement of the wedge pressure. Then, the perfusion was changed to an open mode in presence and absence of L-NMMA, respectively. Dose-response curves using hepatic arterial infusion of 6 consecutive doses of the α_1_-agonist methoxamine (10^–6^ to 3x10^–4^ M; interval time two minutes; Sigma Chemicals Co., Germany) were constructed.

Global liver viability was assessed by gross appearance of the liver, obtention of stable perfusion curves and bile production during the stabilization period (>0.4 μl/min per gr of liver). After the experiment liver and spleen were removed and weighed.

### Calculation of vascular resistances

According to Ohm’s Law, hepatic arterial vascular resistance (HAR) was calculated from the hepatic arterial flow and the hepatic arterial perfusion pressure. Portal venous vascular resistance (PVR) was calculated from the portal venous perfusion pressure and portal venous flow. Sinusoidal vascular resistance (SVR) was calculated from the wedge pressure and the total flow, i.e. portal venous plus hepatic arterial flow.

### Western Blot and Immunohistochemistry

Additional rats were used for tissue collection. After exsanguination of the animal, the hepatic artery and the liver were removed and immediately frozen and stored at-80°C. Samples were homogenized and lysated using 2% Triton-x-100, NaCl, EDTA, EGTA and protease inhibitor. Protein content in the supernatants was quantified using the BCA method with bovine serum albumin as standard. The supernatants were subjected to the SDS-PAGE gel electrophoresis of protein (arteries: 25 μg; liver: 50 μg), and Western blotting was performed using antibodies that recognized parameters for endothelial function, i.e. eNOS, and parameters for intrahepatic hypoxia, i.e. HIF-1alpha (Santa Cruz Biotechnology Inc., Germany). Enhanced chemiluminescence was used for protein detection. Intensity of the bands corresponding to the protein of interest was measured using densitometry.

Immunohistochemistry from liver tissues were performed using antibodies (Santa Cruz Biotechnology Inc., Germany) that recognized phospho-eNOS and HIF-1 alpha. Briefly, sections were treated with PBS containing 0.3% hydrogen peroxide, 5% BSA, 0,5% Tween 20 and incubated with the primary antibody. Bound antibodies were visualized using diaminobenzidine as the chromogen, and slides were then counterstained with hematoxylin solution for 10 min before being mounted and examined using light microscopy (Keyence Deutschland GmbH, Germany, Biozero BZ-8000K). For the negative control, PBS was used instead of the primary antibody. Semiquantitative measurement of the density was done by using Image J.

### Measurement of Remodeling in hepatic arteries

Liver tissue samples from rats described above for Western Blot and Immunohistochemistry from the right hepatic lobe were carefully excised and stained using hematoxylin and eosin (HE).

Morphometric analyses of arterial vessels from the entire sample were performed using video microscopy (Keyence Deutschland GmbH, Germany, Biozero BZ-8000K). The image was captured and displayed on a computer monitor using an image analysis program (Keyence Deutschland GmbH, Germany, BZ image analysis application). The perimeter of the vessel lumen was measured in every vessel and taken as the circumference (C) of a circle. Lumen-Diameter (LD) was determined from the equation LD = C/π assuming that the cross section of the vessel was circular in vivo [10]. Wall thickness (WT) was measured eight times in every vessel (i.e. every 45°) as the linear distance between endothelium and adventitia and the values were averaged. Hematoxylin positive nuclei (N) were counted in each vessel. In order to compare different vessels, the ratio of wall thickness to diameter (WT/LD) and number of nuclei to diameter (N/LD) were calculated and used for comparison between the different groups [10].

### Statistical Analysis

Data are presented as means ± SEM. U-Mann-Whitney test was used for comparisons of two groups and One-Way-ANOVA for comparison of more than two groups followed by post-hoc Fisher test with Bonferroni correction to detect differences between the groups. Comparison for repeated measurements was assessed using multivariate ANOVA of repeated measurements followed by post-hoc Fisher test with Bonferroni correction to detect differences between groups, p-values ≤0.05 were considered significant.

Logarithmic EC50 (pEC50) concentration of the curve were calculated using Prism 6 (GraphPad Software, Inc., US).

The local Institutional Animal Care committee approved all procedures involving animals (Protocol number: K6IG-22).

## Results

### Bivascular liver perfusion

**Fig 1:**
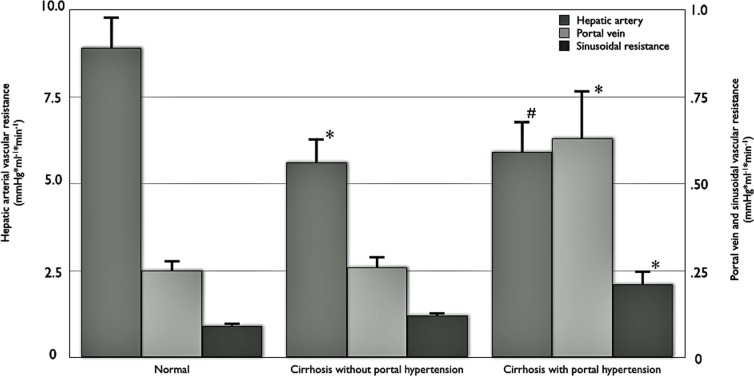
Basal vascular resistances of hepatic artery, portal vein and sinusoidal area in normal (n = 10) and cirrhotic rats with (n = 7) and without elevated portal resistance (n = 6; *p≤0.02 compared to normal; ^#^p = 0.08 compared to normal). Note the ten-fold difference in the scale of the hepatic arterial vascular resistance (left side) and the portal vein/sinusoidal vascular resistance (right side).

Basal vascular resistances of the portal vein, the hepatic artery and the sinusoids in the three different experimental groups are shown in Fig. 1. Animals with ascites (12 weeks of CCl_4_-inhalation) had significantly higher portal vein vascular resistance (0.64±0.14 mmHg*ml^-1^*min^-1^) and sinusoidal vascular resistance (0.21±0.04 mmHg*ml^-1^*min^-1^) compared to normal animals (PVR: 0.25±0.03 mmHg*ml^-1^*min^-1^; SVR: 0.09±0.01 mmHg*ml^-1^*min^-1^) as well as to animals with cirrhosis and without ascites (8 weeks of CCl_4_-inhalation; PVR: 0.26±0.03 mmHg*ml^-1^*min^-1^; SVR: 0.12±0.02 mmHg*ml^-1^*min^-1^; Fig. 1), therefore demonstrating the presence of elevated portal resistance in animals with ascites and the normal portal resistance in animals without ascites. Hepatic arterial vascular resistance in cirrhosis with (5.92±0.92 mmHg*ml^-1^*min^-1^) or without (5.59±0.71 mmHg*ml^-1^*min^-1^) elevated portal resistance was lower compared to normal animals (8.88±0.92 mmHg*ml^-1^*min^-1^; Fig. 1).

**Fig 2:**
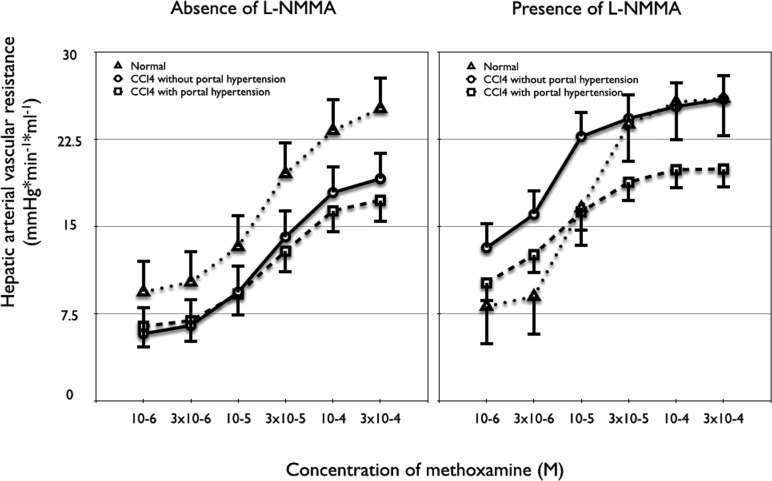
Dose-response curves to methoxamine in absence (left side) and presence (right side) of the nitric oxide synthetase inhibitor L-NMMA. Left side showing a higher hepatic arterial vascular resistance in normal (n = 10) compared to cirrhotic rats with normal portal resistance (n = 6; p = 0.046) and compared to cirrhotic rats with elevated portal resistance (n = 7; p = 0.018). Right side showing no significant differences between hepatic arterial vascular resistance in normal (n = 10) and cirrhotic rats with normal portal resistance (n = 6; 0.321) as well as with elevated portal resistance (n = 7; p = 0.835). Note the difference in maximal vasoconstriction between rats with or without elevated portal resistance/normal as a sign of less smooth muscles in cirrhosis with elevated portal resistance.

The hepatic arteries of cirrhotic rats with elevated portal resistance showed significantly less vasoconstriction (logEC50: 3.73±0.47) in response to methoxamine compared to normal rats (logEC50: 3.65±0.22; Fig. 2; p = 0.018). Similar findings were observed in the cirrhotic rats normal portal resistance, as the dose response curves to methoxamine in these animals revealed less vasoconstriction (logEC50: 3.68±0.37) compared to normal rats (p = 0.046, Fig. 2). However, no difference was observed when comparing both groups of cirrhotic rats, that is normal and elevated portal resistance. Presence of L-NMMA improved the response to methoxamine in cirrhosis with elevated portal resistance (logEC50: 2.46±0.65), so that no differences between dose response curves could be observed compared to normal animals (logEC50: 3.04±0.08). In cirrhosis with normal portal resistance, presence of L-NMMA also normalized the response to methoxamine (logEC50: 2.37±0.22). As shown in Fig. 2, the dose response curves of the three different groups in presence of L-NMMA were not significantly different.

**Fig 3:**
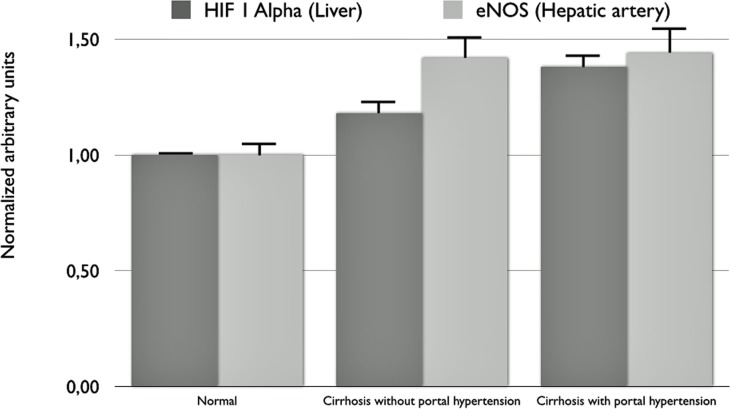
Dose-response curves of methoxamine infusion in the hepatic artery of the portal vein resistance and the sinusoidal vascular resistance in absence (top) or presence (bottom) of L-NMMA. Portal vein and sinusoidal resistance was significantly higher in 12 weeks treated animals compared to normal (PVR: p = 0.0019; SVR: p = 0.007) and 8 weeks (PVR: p = 0.031; SVR not significant different: p = 0.071) treated rats. In presence of L-NMMA only the sinusoidal vascular resistance was different between rats with elevated portal vascular resistance compared to normal (p = 0.003) but not different to the normal portal vascular resistance group (p = 0.094).

The dose response curves of the portal venous vascular resistance during infusion of methoxamine in the hepatic artery showed significant differences when comparing cirrhotic rats with elevated portal resistance with both normal animals (p = 0.019) and cirrhotic rats normal portal resistance (p = 0.031). Again, when L-NMMA was added no differences were observed in the portal venous dose response curves among the three groups (Fig. 3).

The sinusoidal vascular resistance during infusion of methoxamine in the hepatic artery was greater in cirrhotic rats with elevated portal resistance compared with normal (p = 0.007), although no significant difference was observed when comparing to animals with normal vascular resistance (p = 0.071). These results were unchanged in the presence of L-NMMA with significant differences between the curves when comparing cirrhotic with elevated portal vascular resistance and normal rats (p = 0.003) but again no significant difference between cirrhotic rats with and without elevated portal resistance (p = 0.094; Fig. 3).

### Western Blot and Immunhistochemistry

**Fig 4:**
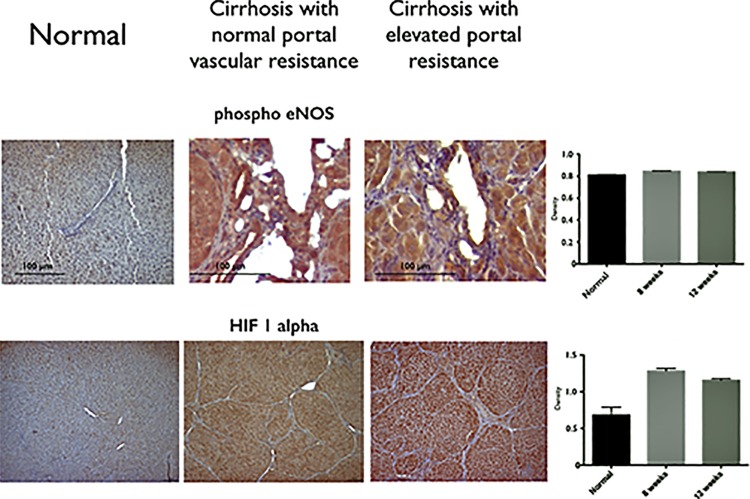
Results of Western Blot showing HIF-1 alpha (liver samples) and eNOS expression (hepatic artery samples). HIF-1 alpha was significantly higher in cirrhotic rats with elevated portal resistance (n = 7; p = 0.05). Rats with normal portal resistance (n = 6; p = 0.66) showed an expression between the other two groups with slightly lower expression of HIF-1 alpha compared to cirrhotic rats with elevated portal resistance and slightly higher expression compared to normal (n = 7; p = 0.73). Expression of eNOS was significantly higher in both, cirrhotic with normal portal resistance (p = 0.03) and with elevated portal resistance (p = 0.02) compared to normal.

**Fig 5:**
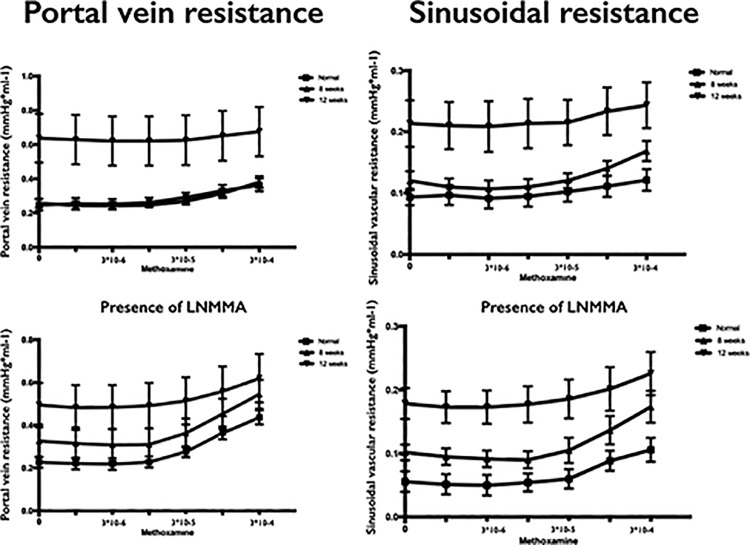
Immunhistochemistry showing the higher expression of phospho-eNOS in hepatic arteries and HIF 1 alpha in livers of cirrhotic rats without or with elevated portal resistance compared to normal animals.

Western Blot and immunhistochemistry showed a higher expression of eNOS in hepatic arteries from cirrhotic rats compared to normals. This difference was observed in rats normal portal resistance (p = 0.03) as well as in rats with elevated portal resistance (p = 0.023, Fig. 4) compared to normal animals. Immunhistochemistry revealed higher expression of phospho-eNOS in the hepatic arteries of cirrhotic livers in animals with normal and elevated portal resistance (Fig. 5) compared to normal rats. Interestingly, HIF-1 alpha expression was higher in cirrhotic livers of rats with elevated portal resistance compared to normal (p<0.05). Cirrhotic rats with normal portal resistance had a higher expression of HIF-1 alpha than normal rats and equal to rats with elevated portal resistance (Fig. 4 and Fig. 5; p<0.001 vs. normal; p = 0.555 vs. cirrhosis with elevated portal resistance).

### Remodeling

The lumen diameter of the hepatic arteries were 57.6±17.7 μm (cross section area [CSA]: 45.25±7.29 μm^2^) in normal animals, 91.3±52.8 μm (CSA: 71.69±15.51 μm^2^) in rats without portal hypertension and 106.0±78.1 μm (CSA: 83.28±26.89 μm^2^) in arteries of cirrhotic rats with portal hypertension. N/LD was significant different between the three groups (p<0.001). N/LD of hepatic arteries in cirrhotic rats with elevated portal resistance was 0.16±0.018 (p = 0.001 vs. normal; p = 0.01 vs. normal portal resistance), in cirrhotic rats with normal portal resistance 0.29±0.034 (p = 0.014 vs. normal) and in normal animals 0.53±0.086. WT/LD in hepatic arteries was significantly different between the three groups (p = 0.002). WT/LD of hepatic arteries in cirrhotic rats with elevated portal resistance was 0.07±0.012 (p = 0.005 vs. normal; p = n.s. vs. normal portal resistance), in cirrhotic rats with normal portal resistance 0.10±0.016 (p = 0.05 vs. normal) and in normal animals 0.21±0.04.

## Discussion

In the present study we demonstrate that in early cirrhosis, hepatic arterial vasodilation is present despite normal portal resistance and that this effect, mediated by nitric oxide, is most likely driven by intrahepatic hypoxia. Despite the presence of subtle structural changes at this stage as shown by the remodeling which is already present, these are not functionally relevant as shown by the perfusion pressure of the hepatic artery, so that nitric oxide is the main factor responsible for the dilatation of the hepatic artery. This suggests that the regulation of the hepatic arterial flow seems to be independent from the changes in the portal venous resistance in cirrhosis.

The liver has a unique vascularization depending on the hepatic artery and the portal vein. Both systems flow into the sinusoid, which is then drained by the hepatic vein. Little attention has been given to the influence of the hepatic artery in the hemodynamic changes that take place in end-stage liver disease, although it is known that hepatic arterial flow progressively increases in patients with cirrhosis as the disease advances [13,14]. Furthermore, it is well established that in the setting of liver transplantation and liver surgery, the hepatic artery is of utmost importance, since inadequate arterial perfusion is associated to liver dysfunction that can even lead to emergency retransplantation [15–17].

We had previously shown that the hepatic artery is dilated in animals with end-stage cirrhosis with ascites [10]. This finding was associated to an over-expression of nitric oxide and remodeling of the wall of the vessel. The mechanisms that regulate this vasodilatation are largely unknown. The hepatic arterial buffer response has been described in normal livers as a compensatory mechanism of the hepatic artery in response to acute changes of portal venous flow [2,4,18]. In the normal liver, the main drive of this mechanism is the maintenance of the total liver blood flow and partial pressure of oxygen in the sinusoids [19,20]. The mechanisms involved in the hepatic arterial vasodilation secondary to the chronic changes of portal venous flow that take place in cirrhosis, are unclear. The development of portal hypertension in cirrhosis leads to a progressive decrease in the perfusion of the sinusoids, so that theoretically there is a reduction of sinusoidal perfusion. On the other hand, cirrhosis is characterized by the development of hypoxia at the sinusoidal level [21]. The presence of hypoxia is considered to be an early phenomenon in cirrhosis induced by inflammatory processes in the liver [22]. Among other changes induced by hypoxia, it increases hypoxia-induced factor-1 (HIF-1) that targets different genes involved in angiogenesis, vascular tone and remodeling [12,22,23]. Taking into account these premises, we aimed to evaluate which mechanism would have a predominant role in cirrhosis.

Interestingly, the animals with cirrhosis had arterial vasodilatation independent from portal resistance, suggesting that the regulation of the hepatic arterial flow is, at least partially, independent from changes in the portal venous circulation. Our results suggest that hypoxia is the main drive for hepatic arterial vasodilatation in cirrhosis. In our experiments, the HIF-1 alpha was lower in normal rats compared to cirrhotic rats. This factor targets genes which encode proteins that play key roles in both immediate and prolonged adaptations to hypoxia. This in turn leads to an increased expression of vasodilatatory factors [12] that could increase hepatic arterial flow independent of portal venous perfusion and pressure [24], such as adenosine. Previous studies have shown that hypoxia leads to an increase of adenosine and a switch of the predominant adenosine receptor from the Adenosine A2 to the Adenosine A1 receptor [11]. Overexpression of the Adenosine A1 receptor is observed in hepatic arteries of cirrhotic rats, and its activation leads in turn to vasodilation and an increase of NO [11,25]. Another possible mechanism involved in the up-regulation of nitric oxide by HIF is Kruppel-like factor 2 (KLF2), which is induced by moderate expression of HIF and does up-regulate the transcription of eNOS [26,27].

Nitric oxide is the main vasodilator of the hepatic artery in end-stage liver cirrhosis [10]. Blockade of nitric oxide during perfusion of the hepatic arteries in cirrhosis with portal hypertension corrects the vasoconstrictive response to normal. Nevertheless, a difference in the maximal response to vasoconstrictor is still detected in the hepatic artery of animals with advanced cirrhosis in comparison to normals due to the remodeling with less smooth muscle in the vessel wall leading to a thinner wall of the vessel [10]. In the present study reversal of the hepatic arterial vasodilation in early cirrhosis without portal hypertension was observed when a nitric oxide inhibitor was added. In contrast to end-stage liver disease, in early cirrhosis normalization of the hepatic arterial resistance was observed, due to less pronounced remodeling in the vessel wall. This further supports that nitric oxide is also the main vasodilator in cirrhosis without portal hypertension.

Nevertheless our study has limitations which are intrinsic to the experimental design. In the perfusion system there is a constant flow. As mentioned above and as shown by other groups, the changes of hepatic arterial flow were related to changes in portal venous flow. In our perfusion system the portal flow is constant, so that perfusion pressure is associated to portal venous resistance. Therefore, acute changes of hepatic arterial flow in response to acute changes in portal venous flow are not measured. In the present study, chronic changes in hepatic arterial resistance with functional and structural changes of the vessel in response to chronic changes of portal venous resistance are measured, although the method only allows evaluation of the intrahepatic portal resistance, without considering other sources of resistance that exist in cirrhosis (i.e collaterals).

In summary, vasodilatation of the hepatic artery is present in cirrhosis with normal and elevated portal resistance suggesting an independent regulation of hepatic arterial flow from portal venous flow in cirrhosis, which is driven by the intrahepatic hypoxia. In this sense, the hepatic arterial vasodilatation would lead to an increase oxygen delivery. Regardless of the presence or absence of elevated portal resistance, the main vasodilator of the hepatic artery is nitric oxide.
